# Apparent Life-Threatening Event following Maternal Use of Temazepam during Labour

**DOI:** 10.1155/2014/650605

**Published:** 2014-06-15

**Authors:** L. Damen, D. H. Visser, S. D. Sie, M. M. van Weissenbruch

**Affiliations:** Division of Neonatology, Department of Pediatrics, VU University Medical Center, P.O. Box 7057, 1007 MB Amsterdam, The Netherlands

## Abstract

Benzodiazepines are one of the most frequently prescribed psychotropic drugs during pregnancy. Despite the fact that these drugs have been in use for a long time, there is still debate about the safety for the developing fetus and neonate. We present a case of a newborn with an apparent life-threatening event shortly after birth following maternal temazepam use during labour and advise to be reserved in prescribing any dose of any kind of benzodiazepine during labour.

## 1. Introduction

Benzodiazepines are one of the most frequently prescribed psychotropic drugs during pregnancy [1]. However, there are still concerns about the safety for the developing fetus. We present a case of a newborn with an apparent life-threatening event shortly after birth following maternal temazepam use during labour.

## 2. Case Presentation

**Figure 1:**
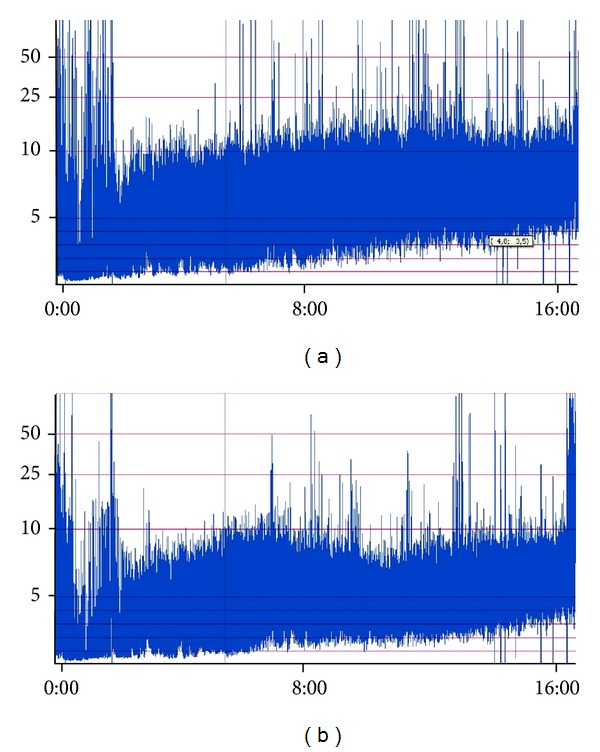
Cerebral function monitoring (CFM) of an infant newborn with an apparent life-threatening event following maternal temazepam use during labour. On *x*-axis time (in hours) since admission on neonatal intensive care unit. On *y*-axis cerebral function of right (b) and left side of the brain expressed in microvoltage (*μ*V). Initially CFM monitoring showed burst suppression and changed to continuous low voltage in 2-hour time. Later on, CFM pattern returned to a discontinuous normal voltage.

A 38-week-old female neonate, born via a spontaneous vaginal delivery guided by a midwife at home, was presented to our Neonatal Intensive Care Unit (NICU) after an episode of apnea, bradycardia, and hypotonia one hour postpartum. The mother, a healthy 30-year-old gravida 3 para 1 had an uneventful pregnancy during which she did not use any medication. Cervix dilation progressed slowly and the midwife was prescribed temazepam 20 mg to prevent maternal exhaustion in the first stage of labour. However, in contrary to what was expected, labour progressed quickly and three and a half hours since temazepam ingestion mother gave birth to a female infant newborn. Apgar Scores 1 and 5 minutes after birth were both 10 out of 10. Birth weight was 3650 gram (+1 SD), length was 34 cm (+1 SD), and head circumference was 34 cm (−1 SD). Breastfeeding was initiated immediately after birth. One hour later the baby was found nonbreathing, pale, and hypotonic on mothers breast. The attending midwife initiated neonatal resuscitation. Chest compressions were started in combination with ventilation because of persisting bradycardia after inflation breaths. Heart frequency normalized and spontaneous breathing returned. She was intubated and transported by the mobile medical team to our hospital in stable condition. Neurologic examination at arrival at the NICU showed a normal muscle tone and symmetric movements of all limbs. Pupils were isocore and reactive to light. Sucking reflex was present, while grasp reflex was absent. Initial blood workup showed normal values for glucose and electrolytes but a venous serum lactate of 8.6 mmol/L. Capillary blood gas showed a pH of 7.35, pCO_2_ of 31 mmHg, HCO_3_^−^ of 16.9 mmol/L, and base excess of −7.4. Investigation for inborn errors of metabolism did not show any abnormalities. Blood culture was taken and antimicrobial therapy was started empirically. Culture did not show any microorganisms and microbial therapy was stopped 48 hours after treatment initiation. Brain ultrasound did not show any abnormalities. Initially cerebral function monitoring (CFM) showed burst suppression and changed to continuous low voltage without signs of epileptic activity in 2-hour time (Figure 1). Supportive therapy with mechanical ventilation could be stopped several hours after admission and CFM pattern returned to a discontinuous normal voltage. Screening of urine was positive for benzodiazepines and a supratherapeutic temazepam plasma level of 145 *μ*g/L (20–100 *μ*g/L) was found six hours since maternal temazepam intake. Three days later the neonate was fully recovered and could be discharged from the NICU.

## 3. Discussion

Benzodiazepines have hypnotic, anxiolytic, anticonvulsive, and muscle relaxant abilities. They are mainly used for sleeping and anxiety disorders. In addition, they are used as anticonvulsant and as hypnotic drug during medical treatment [2]. Benzodiazepines cross the human placenta freely and thereby reach the fetus [1, 3, 4]. Despite the fact that these drugs have been in use for a long time, there is still debate about the safety for the developing fetus and neonate. Most studies evaluating neonatal effects of benzodiazepine use during late pregnancy concern diazepam [5–7]. No studies are published about temazepam use during labour. Temazepam is a short-acting benzodiazepine with a half-life of 7–11 hours and oral bioavailability of >90% and reaches peak plasma concentrations in 1-2 hours, while diazepam is a long-acting benzodiazepine with a half-life of 20–48 hours. However, temazepam has structural similarities to diazepam and therefore is thought to act in a similar way [1]. Both drugs enhance the effect of *γ*-aminobutyric acid (GABA) at the GABA_A_ receptor causing an inhibitory effect on neurotransmission by diminishing the chance of a successful action potential resulting in the pharmacodynamic effects described before. Temazepam is metabolized by the liver through glucuronidation by uridine diphosphate glucuronosyltransferase (UGT) and excreted through the kidneys [2]. In newborns, the liver and kidney functions are still in a stage of development resulting in delayed elimination of benzodiazepines and therefore accumulation may occur.

When benzodiazepines are used during the last trimester of pregnancy a floppy infant syndrome and withdrawal syndrome have been described [8]. Floppy infant syndrome has been thought to be a result of benzodiazepine intoxication with symptoms like hypotonia, hypothermia, lethargy, respiratory difficulties, and sucking difficulties [3, 8], while withdrawal symptoms constitute irritability and hypertonia [3, 8, 9]. Nevertheless, these syndromes are often difficult to distinguish and symptoms frequently overlap. Furthermore, studies have shown an association between benzodiazepine use and low 5-minute Apgar Score, admission to a NICU, and respiratory distress syndrome, especially when larger doses of diazepam (>30 mg) are used during labour [6, 9, 10]. In addition episodes of primary or secondary apnea and apneic spells have been described [7]. Laegreid et al. found significantly more neurologic symptoms (depression and agitation) on days 2 and 3 after delivery in infants whose mothers have used benzodiazepines throughout pregnancy [4]. Others found significant lower mean rectal temperature within the first three hours after birth in neonates whose mothers had received diazepam during labour [5–7, 10].

This case presents a newborn with an apparent life-threatening event shortly after birth most probably caused by maternal temazepam use during labour. A single dose of 20 mg temazepam resulted in a supratherapeutic plasma level in the newborn. Since 20 mg temazepam is considered a small dose, we advise to be reserved in prescribing any dose of any kind of benzodiazepine during labour.
